# Comparing adult renal stem cell identification, characterization and applications

**DOI:** 10.1186/s12929-017-0339-7

**Published:** 2017-05-16

**Authors:** Jennifer Huling, James J. Yoo

**Affiliations:** Wake Forest Institute for Regenerative Medicine, Wake Forest School of Medicine, Medical Center Boulevard, Winston-Salem, 27157 USA

**Keywords:** Renal, Stem Cell, Regenerative Medicine

## Abstract

Despite growing interest and effort, a consensus has yet to be reached in regards to the identification of adult renal stem cells. Organ complexity and low turnover of renal cells has made stem cell identification difficult and lead to the investigation of multiple possible populations. In this review, we summarize the work that has been done toward finding and characterizing an adult renal stem cell population. In addition to giving a general overview of what has been done, we aim to highlight the variation in methods and outcomes. The methods used to locate potential stem cell populations can vary widely, but even within the relatively standard practice of BrdU labeling of slowly dividing cells, there are significant differences in protocols and results. Additional diversity exists in cell marker profiles and apparent differentiation potential seen in potential stem cell sources. Cataloging the variety of methods and outcomes seen so far may help to streamline future investigation and stear the field toward consensus. But even without firmly defined populations, the application of renal stem cells holds tantalizing potential. Populations of highly proliferative, multipotent cells of renal origin show the ability to engraft in injured kidneys, mitigate functional loss and occasionally show the ability to generate nephrons *de novo*. The progress toward regenerative medicine applications is also summarized.

## Background

The kidneys are complex and critical organs. Even with considerable redundancy built in, the slow deterioration of renal tissue over time can lead to life threatening complications. Unlike some species, humans cannot create new nephrons as adults, but instead rely on replacing individual lost cells [1, 2]. Unfortunately, this regenerative capacity is limited and age or disease can cause renal function to drop below critical levels. Chronic kidney disease (CKD) affects more than 26 million Americans [3]. Existing treatment options for CKD are limited. Dialysis artificially replaces some lost functions, but does not treat the underlying problem [4]. Whole organ transplants are curative, but the supply of organs is low and long term survival rates are less than 40% [4, 5].

Tissue engineering and regenerative medicine research seeks better treatment alternatives through the application of adult renal stem cells. Stem cells sourced from the adult kidney have the potential benefits of improved renal engraftment and differentiation and can be utilized in autologous therapies. Work has been done to identify renal stem cells, but so far, no consensus has been reached.

The goal of this review is to assess the existing body of work related to identifying and applying renal stem cells. In each case, potential renal stem cells populations are evaluated on criteria often used to define and characterize adult stem cell populations, namely self-renewal, colony forming ability, molecular marker profiles and multipotency [6]. Special attention was given to differences in method and variations in results. The field has aquired a good general base of knowledge, but scrutiny of the techniques and outcomes will be necessary to move toward a unified explanation of renal regeneration and the role of organ-specific stem cells in the kidney.

The kidney is an anatomically complex organ with a huge variety of cell types and cell environments [7]. Because of this, we will discuss potential stem cell sources based on anatomical origin, namely from the Bowman’s capsule, the renal papilla and the nephron tubules. Research into renal stem cells may help elucidate mechanisms of renal homeostasis and healing, but the ultimate goal is to utilize those stem cells for clinical treatments. To this end, the review includes a summary of the applications of renal stem cells in animal models of acute kidney disease and the *de novo* formation of renal tubule structures in 3D culture.

## Renal stem cells in the Bowman’s capsule

Podocytes are a specialized type of epithelial cell that encase the glomerular capillaries with interdigitated foot processes to regulate filtration into the nephron [8]. Interestingly, podocytes occasionally detach and are excreted in the urine [9, 10]. Generally, podocytes are not considered to be mitotically active [8]. Therefore, a source of renal stem cells capable of replacing the lost podocytes is through to exist.

The Bowman’s capsule contains a subset of parietal epithelial cells (PECs) that are believed to be a source of adult stem cells in the kidney. Staining of cortical renal tissue revealed cells that co-expressed the common stem cell marker CD133 and the renal embryonic cell marker CD24 [11]. A large CD133+/CD24+ population in developing embryonic kidney displays self-renewing and multipotent characteristics, but this population decreases in prevalence as development progresses [12]. In adult human kidneys, CD24+/CD133+ cells remain scattered throughout the tubules and the urinary pole of Bowman’s capsule [11, 13, 14]. CD133+/CD24+ cells isolated from Bowman’s capsule were found to express the stem cell markers CD106, CD105, CD54, and CD44 [13, 14]. This potential stem cell population was negative for the podocyte markers PDX, nephrin, podocin, synaptopodin, WT-1, and the tubule proteins EMA-1, THG, LTA, and AP, which indicates that the cell population was not yet fully committed to a specific renal linage [13, 14]. CD133+/CD24+ cells from Bowman’s capsule were labeled as adult parietal epithelial multipotent progenitors (APEMP) [13]. One measure of stemness was demonstrated by clone formation in vitro [13, 14]. This same cell population is thought to occasionally end up in the urine of patients with glomerular diseases [15]. They are rare, but can be cultured into usable numbers and perform similarly to cells isolated from whole tissue.

Typically, adult stem cells are thought to proliferate very slowly in healthy tissue, allowing for identification with 5-bromo-2-deoxyuridine (BrdU) in pulse-chase experiments [16]. Briefly, BrdU is incorporated into dividing cells during the pulse phase, but further division during the chase phase quickly dilutes the BrdU. Ideally, only cells that divide infrequently remain labeled with the BrdU. These cells are called as label retaining cells (LRCs) and are often the first target when searching for a new adult stem cell population [16, 17]. To confirm that APEMPs were LRCs, rat kidneys were labeled for 14 days with a BrdU pulse and chased out to 14 weeks. LRCs were confirmed at the urinary pole of glomeruli [18].

**Fig. 1:**
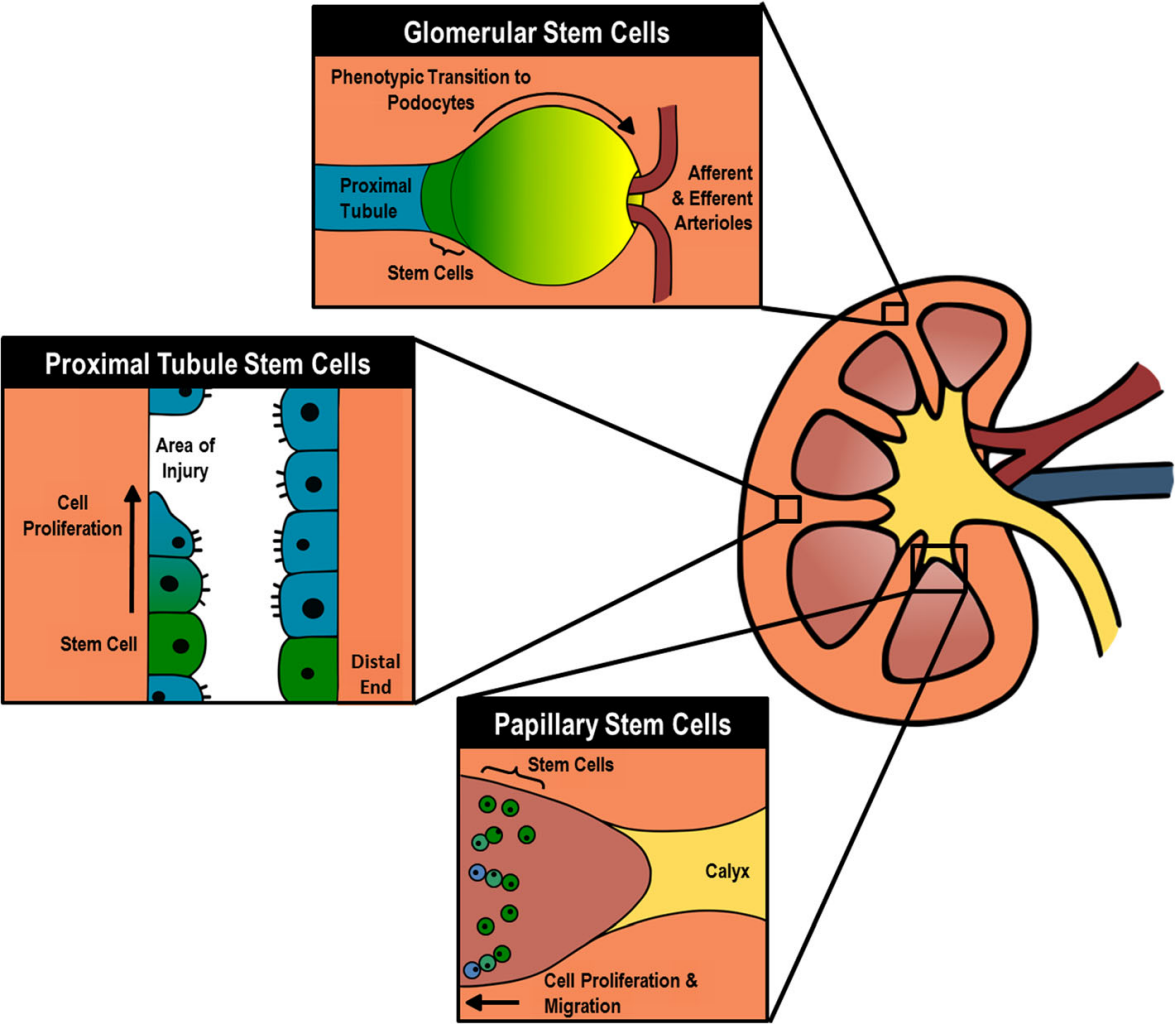
Diagram showing some of the studied areas which may contain renal stem cells. Green areas or objects represent stem cells. In the glomerulus, stem cells are thought to reside in at the urinary pole of the Bowmans capsule and differentation into podocytes. Proximal tubules are thought to have an ischemia-resistant population of stem cells at their distal end. These cells proliferate to fill in areas of damage. In the base of the papilla, there is thought to be a population of stem cells which response to damage by producing cells which may migrate to the rest of the tissue

APEMP cells are located at the urinary pole of Bowman’s capsule which is continuous with the layer of podocytes surrounding the glomerular capillaries, providing the APEMP cells unbarred access to the podocytes’ location. A gradient population of cells has been found to connect the undifferentiated APEMP cells at the urinary pole with fully differentiated podocytes at the base of the vascular pole (Fig. 1) [14]. As cells move from the urinary pole to the vascular pole, they begin to acquire podocyte traits and lose stem cell traits. This is reflected in a morphological transition, noted in many animal species and humans [18, 19]. Cell marker analysis has shown that as cells move closer to the vascular pole they first acquire the podocyte marker PDX followed by loss of the stem cell markers CD24 and CD133, which corresponds to a loss of self-renewing and differentiation capabilities in the cells in vitro [14]. Transgenic mice with labeled PECs can be used to demonstrate differentiation into podocytes [18]. In one case, lineage tracing of PAX2 cells in mice showed direct differentiation from progenitor cells into podocytes and linked podocyte regeneration with better injury recovery [20].

The differentiation potential of APEMP cells has also been examined in vitro. These cells have been differentiated in culture to display characteristics of proximal and distal tubule cells as well as podocytes [13, 14]. In addition, non-renal differentiation capacity has been demonstrated in the form of osteogenic, adipogenic, and neurogenic differentiation [13].

## Renal stem cells in the papilla

The first evidence of a stem cell population in the papilla came from BrdU pulse-chase experiments in young rodents in which a 3.5 day BrdU pulse followed for 2-3 months identified LRCs that occurred infrequently in cortical structures and appeared densely in the papilla [21]. Further examination of this group of LRCs showed an increased density at the base of the papilla [22]. If these LRCs represented a stem cell population, having the cells positioned closer to the cortex could be advantageous to decrease migration distance. In addition to having a gradient distribution along the papilla, the LRCs appeared both as interstitial cells and integrated within collecting ducts [21, 22]. Among the LRCs located at the base of the papilla, a much higher percentage of them were interstitial, which may indicate that these cells represent the actual stem cell population while the tubular cells may play a role in normal maintenance [22]. Supporting this hypothesis is the fact that only interstitial papillary LRCs express nestin, a stem cell marker [22, 23]. LRC labeling with EdU (5-ethynyl-2-deoxyuridine) has shown a similar high LRC density in the papilla rather than the cortex [24]. These results are seemingly at odds with the experiments showing LRCs in the cortex.

Despite the widespread use of BrdU labeling for the identification of possible stem cell populations, the technique has limitations. If adult stem cells are dividing infrequently, they may not divide during the pulse and would not be labeled. In addition, the chase period needs to be sufficiently long to allow dilution of the dye in normally proliferating adult cells. This can be a particularly delicate balance to achieve in the kidney which has normally low mitotic index. Mice labeled within days after birth were observed to have more LRC in the papilla, while adult mice labeled in the same manner showed a distribution skewed towards the medulla and cortex [25]. BrdU protocols applied to the kidney vary significantly, with few showing supporting data for their pulse-chase timing [26]. A suggested alternative to BrdU labeling is the use of transgenic mice that can be triggered to ubiquitously express GFP in their kidneys, which is diluted with multiple cell divisions. This technique avoids the possibility of missing very slowing replicating cells during the labeling phase and has been shown to more accurately label certain stem cell populations [27, 28]. Oliver et al. demonstrated similar renal LRCs in both rats pulsed with BrdU and transgenic GFP-expresssing mice [22, 29].

In addition to using BrdU labeling, several markers have been used to identify and define the stem cell population in the papilla. Nestin, CD133, CD24a, CXCR7, and Pax2 have all been reported as potential defining markers of the papillary stem cell in rodents [21, 30]. Expression of telomerase reverse transcriptase has been shown to be highest in the renal papilla of mice, which also supports the presence of stem cells [31]. Limited studies of self-renewal capabilities in papillary LRCs have been performed. One study produced single-cell clones from isolated LRCs, but these cells gave rise to heterogeneous populations with morphology that was very dependent on culture conditions [21]. Nestin has been a common marker for identifying stem cells in the papilla, but one group claims that it can be used to identify mesenchymal stem cells resident in the kidney [32]. Nestin+ cells isolated from whole kidney where characterized to have a mix of renal and mesenchymal markers. This would seem to contradict work that used a very similar method of isolating cells but reported different cells phenotypes [30]. Both groups collected GFP+ cells from Nestin-GFP transgenic mice.

In vitro culture of isolated papillary LRCs from rodents has shown pluripotent potential [21]. Culture in serum-free media led to the formation of cell aggregates reminiscent of neurospheres. These cells occasionally expressed the neuronal markers of nestin and class III-beta-tubulin. Additionally, the LRCs could be cultured to express the epithelial marker ZO-1 in a cell monolayer or to become spindle-like and express alpha-smooth muscle actin. There is some early evidence that nestin-positive cells in the papilla participate in angiogenesis and may differentiate to endothelial-like cells [30].

While there is some belief that stem cells found in the papilla are involved in normal maintenance of the nearby tubules and collecting ducts, these cells also appear to respond to renal ischemic injury [21]. Ischemic injury is followed by a reduction of BrdU and nestin labeled populations [21, 30]. The reason for the reduction of cells is not completely understood. Papillary LRCs can migrate within the papilla, but do not seem to directly migrate into the cortex [21, 22]. Instead, LRCs and nestin-positive cells both show a tendency to migrate toward the base of the papilla and begin dividing in response to ischemia [21, 22, 30]. Chains of dividing cells can be seen at the base of the renal papilla and may indicate that the LRCs produce transit-amplifying daughter cells after injury [22]. Individual cell migration toward the cortex has been noted ex vivo in response to ischemic conditions [30]. It is unclear whether direct migration of the LRCs or migration of their daughter cells is the primary mechanism of repair. A recent lineage-tracing study identified a subpopulation of LRC’s cells in the base of the papilla that express protrudin [29]. Interestingly, these cells replicate at a very slow rate and do not participate in homeostatic cell replacement or cell repair after mild injury. Above a certain threshold of ischemic injury, they begin to replicate and the resulting cells are found in tubules only in the medulla. The role of stem cells in the kidney may be complicated, involving multiple resident cells sources which participate in kidney regeneration only under specific circumstances.

Some notable differences between rodents and humans have arisen when trying to confirm a potential stem cell population in human kidneys. In human renal tissue, CD133+ cells have been confirmed throughout the kidney, but in the medulla they appear specifically in the portions of the loops of Henle reaching into the papilla. It was noted that while these CD133+ positive cells were integrated into the tubule, they were morphologically and phenotypically different from the surrounding tubule cells [33, 34]. This cell population in humans has been noted to express nestin, CD73, CD29, CD44, CD146, SSEA-4, cytokeratin, vimentin, Pax2, Six1, Six-2, c-Myc, Klf4, and Oct-4. The presence of Oct-4 contradicts the work in the mouse model, which did not detect Oct-4 in the papillary stem cell population [22]. In addition, the probable papillary stem cell population in rodents appears primarily in the interstitium as well as the collecting ducts and loop of Henle [29]. It is not clear why the papillary stem cell-like population resides in the papillary interstitium in rodents and in the loops of Henle in humans, but it may be tied to anatomical differences between species. Rodents have a single renal papilla and humans are multipapillate [35]. Perhaps some difference during embryonic development of the papillary structures dictates the unique final locations.

Aside from location differences, human papillary stem cells have shown similar behavior to those isolated from rodents. Human papillary CD133+ cells form colonies in culture and can be differentiated into tubulogenic and neurogenic lineages [33, 34]. It is unclear if the cells found in rodents and humans represent two distinct populations or the same cell type in slightly different niches.

The renal papilla itself is a good candidate for a stem cell niche environment. Generally, the niche environment that supports adult stem cells helps to protect the cells and keep them in a prolonged stem state. The renal papilla is unique in the kidney because of its hypoxic and hyperosmotic environment. More than one known adult stem cell population resides in an hypoxic environment [36, 37]. Hypoxia is thought to help regulate signals within a cell that keep it from differentiating. In human CD133+ cells, it has been shown that hypoxia regulates Oct4a, a marker of stemness [34, 38]. Hypoxia increases Oct4a expression and downregulates miR-145 expression, which, in turn, would downregulate Oct4a and correlates with lowered proliferation [34]. Therefore, CD133+ cells may rely on a hypoxic environment to maintain stemness. In addition, the papilla has been shown to be protected from changes in oxygen concentration and experience the lowest amount of cell death in response to ischemia [21].

In humans, the solute concentrations in the renal medulla can range from 300 to 1,200 mOsM [7, 39]. While this may be a harsh environment for acute exposure to cells, there is evidence that cells with prolonged exposure to hyperosmotic environments will adapt and became more resistant to osmotic changes and other harsh environmental factors [39]. This has been shown to be partly related to a reduction in DNA replication [40]. In many ways, the papilla provides an ideal environment that is protected from damage and conducive to maintaining slowly replicating populations.

## Renal stem cells from tubular origins

BrdU incorporation in adult rat kidneys has shown the presence of LRCs in some cortex tubules [26, 41]. A BrdU pulse for 7 days in 7 weeks old rats was followed with staining 2 weeks later. LRCs were found primarily in the proximal tubules, with less frequent appearance in the distal tubules and collecting ducts. No LRCs were found in the glomeruli or papilla using this protocol, contradicting other studies [18, 21, 22]. These LRCs were shown to proliferate in response to ischemic injury, often appearing in pairs 24 hrs after injury [26, 41]. Staining for the mesenchymal marker vimentin showed no expression in LRCs in healthy kidneys, but vimentin was expressed in LRCs after injury [41, 42]. In addition, pairs of LRCs induced by injury showed one cell expressing vimentin while the other did not, supporting the idea of asymmetric stem cell division [41].

The S3 segment of the proximal tubule in rats has been the most studied tubular segment in the search for renal stem cells. The region located near the corticomedullary junction tends to experience more damage from ischemic injury than other segments of the nephron, which makes this a practical place for a stem cell population [43]. Inducing tubular damage along the S3 segment of the tubule induces vimentin expression and proliferation in some of the remaining S3 tubular cells as they begin to repair the damage [42, 44]. After injury, the proximal three-quarters of the S3 segment incurs the most damage, leaving the highest density of surviving and proliferating cells at the distal end of the S3 segment that is nearer to the medulla [42]. The most distal quarter of the S3 segment seems resistant to injury that causes severe damage to the rest of the segment. It is thought that if there is an ultimate source of stem cells, then they may reside there. The location would protect them from damage and allow easy access to a commonly damaged area of the nephron. Morphologically different cells have been noted near the distal end of the S3 segment, having a cuboidal shape, and lacking the typical brush border [42].

Because of the complicated nature of healing and cell proliferation in the renal cortex, it remains difficult to define a specific subpopulation of stem cells. In rats, little progress has been made in identifying the molecular markers associated with the cells that are poised to replicate after injury. LRCs have been isolated from the kidney cortex of rats and grown in culture. They were shown to be very responsive to their culture environment and could be made to express mesenchymal markers like vimentin or markers of various renal tubules [26]. Culture of microdissected S3 tubules from rats yielded a cell line with stem cell-like qualities [45]. The cell line expressed the stem cell markers vimentin, c-Met, Sca-1, c-kit, Pax-2, and the neural stem cell marker Musashi-1. The cells could be differentiated to form different tubule phenotypes and incorporated into renal tubules when injected into injured kidneys [45].

Gupta et al. isolated stem cells from whole rat kidneys, which they believe originated from the proximal tubule. Colony-forming cells were selected from a plating of the heterogeneous cell suspensions [46]. Very rare cells were able to form clones with extremely high doubling abilities and a possible differentiation capacity for all three germ layers. Interestingly, the cells displayed stem cell-like characteristics and could be engrafted into tubules when injected into injured kidneys, but the phenotypic profile of these cells varied significantly from other studies. While the isolated cells were found to express vimentin, Oct4, and Pax-2, they did not express CD133 or CD106. This conflicts with results from other papers where potential stem cells with those markers were isolated; thus, the importance of isolation and culture conditions for renal stem cells can be inferred. The same group examined alternative markers for renal stem cells by identifying a potential population based on Sall1 expression [47]. In this study, Sall1-expressing cells at the cortical medullary junction in mice proliferated in response to renal injury.

More recent work has been focused on identification of a proximal tubule-based stem cell source in human kidneys. Aldehyde dehydrogenase (ALDH) is thought to play a role in stem cell maintenance; therefore, Lindgren et al. isolated a population of cells with elevated ALDH activity from human kidney cortex [48]. Whole genome expression profiling of the ALDH^high^ cells showed a correlation with previously examined markers such as CD133 and CD24. Staining for these markers in human kidney showed co-localization in cells of the proximal tubule and Bowman’s capsule. The CD133+/CD24+ cells were noted to exist in proximal tubules in general and were sometimes seen in the creases formed in the convoluted proximal tubules. Contradicting similar work in rodents, vimentin staining was co-expressed alongside CD133 and CD24 in healthy tissue. Expression profiles of the ALDH^high^ fraction revealed upregulation of proteins thought to render these cells resistant to apoptotic stimuli; moreover, these expression patterns are similar to other known stem cells. The general phenotypic profile has been confirmed in cortical cells, which showed co-expression of CD133 and CD24 in proximal tubules cells and Bowman’s capsule as well as occasional convoluted distal tubules and the collecting ducts [11, 49]. CD106 was used to differentiate between tubule stem cells (CD133+/CD24+/CD106-) and those in Bowman’s capsule (CD133+/CD24+/CD106+) [11]. Isolated CD133+/CD24+/CD106- cells from human proximal tubules showed self-renewal and differentiated into tubule cell types.

Recent work has drawn parallels between the human stem cell populations defined in the PECs of Bowman’s capsule and evidence of a stem cell population in cortical tubules. In depth immunohistochemistry of CD133+ cells in the cortex clearly confirmed the presence of stem cells in Bowman’s capsule, as expected, and that there were scattered CD133+ cells in the proximal tubules [48]. Additional staining showed co-localization of CD24, vimentin, BCL2, KRT7, KRT19, and MYOF in both populations of CD133+ cells [48]. Further studies by Angelotti et al. aimed to determine methods to distinguish between CD133+/CD24+ in the glomeruli and those in the tubules. Importantly, the CD133+/CD24+ cells in Bowman’s capsule expressed CD106; thus, this marker could be used to differentiate between the two very similar populations [11]. Further analysis showed that CD133+/CD24+/CD106+ cells had slightly higher proliferation and differentiation potentials and were able to differentiate into podocytes. In vitro, all cortical CD133+/CD24+ cells better withstood damage from hemoglobin exposure than CD133/CD24- cells [11].

CD133 is a commonly used marker for stem cells. But it is important to note that in the context of stem cell identification antibodies are detecting the AC133 epitope on the membrane protein which is specific to undifferentiated cells in humans [50]. CD133 is also commonly expressed in adult cells, particularly epithelial cells, as shown by presence of mRNA and staining with polyclonal antibodies [51]. The difference is thought to be in the glycosylation of the extracellular portion of the protein depending on differentiation state [50]. Glycosylation independent staining with anti-CD133 polyclonal antibodies could lead to misinterpretation of stem cell populations [52]. CD133+ stem cell identification in the human kidney utilizes CD133/1 or CD133/2 monoclonal antibodies. One study showed co-localization of both monoclonal antibody types in PECs in Bowman’s capsule [13]. In the human kidney, CD133+ cells have been located most often in the Bowman’s capsule, occasionally in tubule cells, and rarely in the interstitium [11, 13, 14, 48]. Interestingly, one study reported CD133 only in clusters of interstitial cells in human kidneys [53].

The mechanisms of recovery after tubular damage are complicated and controversial. Several mechanisms for tubule repair after injury have been proposed [54]. An increasing amount of evidence supports the idea that surviving tubule epithelial cells undergo an epithelial-to-mesenchymal transition, and the dedifferentiated cells then proliferate to repopulate the damaged area and then differentiate back into epithelial cells. This model is supported by the observation of injury-induced expression of vimentin in proximal tubules cells and some developmental genes like Pax2 [41, 42, 53]. Cell linage-tracing work in transgenic mice has supported the idea that injury recovery in the proximal tubule does not involve LRCs and has shown differentiated cells to be proliferative and responsible for repair [55–58]. One work performed linage tracing of rare Sox9 expressing cells in the proximal tubule, which could potentially represent a resident stem cell population [59]. They showed that these resident Sox9+ cells had some, but relatively little contribution to repair after injury. Instead *de novo* expression of Sox9 was seen in highly proliferative cells during recovery. This again supports the idea that differentiated cells are responsible for recover after injury, but leaves the door open for adult stem cells that contribute to homeostasis.

External stem cell sources have also been considered as a mechanism of repair as some investigators have suggested that bone marrow-derived stem cells could migrate to injured kidneys and differentiate into tubules cells [60, 61]. Conversely, other investigators have suggested that direct replacement of renal epithelial cells does not involve bone marrow-derived cells, but instead those bone marrow-derived cells may engraft and provide paracrine effects [62–64]. Still another proposed mechanism for repair is through the activation of a resident adult stem cell population, which is supported by much of the work described previously, but the dispute remains as to the existence of a renal stem cell.

Much of the work to define a stem cell population in the kidney started with groups searching for known stem markers and then isolating and further analysing cell populations with those markers. Working in the opposite direction, citing the idea that markers are not 100% accurate, Bombelli et al isolated a potential stem cell population based off of self-renewal capabilities [65]. Using sphere-forming methods of culture, they found a population of human renal cells that was able to form nephrospheres. Sphere-based culture has been seen in other renal stem cell isolation attempts and is commonly used in the culture of other adult stem cells like neuronal stem cells and mesenchymal stem cells [66]. The cells giving rise to nephrospheres were identified by maintainance of PKH labeling. So called, PKH^high^ cells demonstrated the asymmetric cell division and longterm passaging potential expected from a stem cell. The PKH^high^ cells showed differentiation potential toward proximal and distal tubules, podocytes and endothelial cells. The cells showed a limited direct regenerative capacity in vivo. As the cells were isolated from bulk human tissue, and do not yet have a distinct phenotype, their origin within the kidney is unknown. Interestingly, a similar attempt at spheroid culture of adult renal cells yielded spheroids that formed through aggregation rather than clonal growth and which supported the idea of dedifferentiation of cells into a stem-like state, rather than the conditional isolation of a stem cell population [67].

The common prevailing dogma in regenerative medicine is that every tissue types has a source of adult stem or progenitor cells responsible for supplying new cells during homeostasis and healing. A summary of the efforts to define and characterize renal stem cells can be seen in Table 1. Several factors confound our ability to point to such a population in kidneys. Kidneys are an extremely complex organ, comprised of more than 25 different cells types. Developmentally, the renal tissue goes through multiple developmental phases and stems from multiple embryonic sources, making its relationship with embryonic stem cells sources complex [68]. Adult kidneys are divided anatomically into distinct regions, with tubular features that are segregated within regions and some that span multiple regions. The normal mitotic activity in renal tissue is very low, making normal homeostatic activity difficult to observe. Under injuries conditions, different parts of the organ see more damage than others depending on the type of injury. All of this complexity and variability makes identifying an adult stem cell population difficult, especially when added to the variability inherent in researching a relatively new area of study. This becomes obvious in conflicting BrdU labeling results and conflicting marker staining and expression. Different culture conditions and media formulations may play a part as renal cell behavior seems strongly influence by growth factors and matrix components [26, 69–71].

**Table 1:** Summary of renal stem cell isolation papers

Sources	Species	Location	Stem cell population markers	BrdU LRC identification	Colony formation	Demonstrated differentiation potential						
						Epithelial	Podocyte	Osteogenic	Adipogenic	Neurogenic	Endothelial	Hepatic
[18]	R/M	Bowman’s capsule		X			X					
[13]	H	Bowman’s capsule	CD133, CD24, CD106, CD105, CD54, CD44		X	X		X	X	X		
[14]	H	Bowman’s capsule	CD133, CD24, Bmi-1		X	X	X					
[11]	H	Bowman’s capsule	CD133, CD24, CD106, vimentin		X	X	X					
		Proximal tubule	CD133, CD24, vimentin		X	X						
[26, 41]	R	Proximal tubules		X		X						
[46]	R	Proximal tubules	CD90, CD44, vimentin, Oct4, Pax-2		X					X	X	X
[45, 69]	R	S3 segment proximal tubule	Mosahi-1, vimentin, c-met, sca-1, c-kit, pax-2, GDNF, WT-1, wnt-4		X							
[53]	H	Cortex	CD133, Pax-2, CD73, CD29		X	X					X	
[48]	H	Proximal tubules	CD133, CD42, vimentin, BCL2, KRT7, KRT19, MYOF									
[21]	R/M	Papilla		X	X	X				X		
[30]	M	Papilla	Nestin								X	
[22]	R/M	Papilla	CD133, CD24a	X								
[33]	H	Papillary Loop of Henle	CD133, nestin, SSEA4, Nanog, Sox2		X	X				X		
[34]	H	Papillary Loop of Henle	CD133, Nestin, CD73, CD29, CD44, CD146, SSEA4, Cytokeratin, Vimentin, Pax-2, Six1, Six2, Oct4, c-Myc, Klf4		X	X						
[65]	H	Unspecific	CD133		X	X	X				X	

Rat (*R*), Mouse (*M*), Human (*H*). The table is organized by the stem cell location within the kidney. The large variability between identified cell markers and differentiation potential is clear. X’s indicate positive occurrences of the corresponding column description. Blank spaces do not imply negative results

## Application of adult renal stem cells

The hunt for renal stem cells began out of a desire to better understand how kidneys regenerate during homeostasis and healing. Recent years have brought a sharp increase in the number of papers exploring numerous methods for finding and defining renal stem cells. There has been substantial progress, but the area of research has yet to reach any kind of unified consensus. We may not be able to point directly to one or many standarized populations, but we can begin to move forward by exploring possible applications. A proliferative, multipotent renal cell source may prove usefull even before its normal physiologic role is completely understood. In this section, the translational potential of renal stem cells will be discussed. In vitro and in vivo regenerative medicine and tissue engineering examples will be compared and summarized. Most examples utilize cells isolated under parameters discussed earlier in the review.

We are currently in need of new treatment options for CKD. Regenerative medicine and tissue engineering are exploring treatment options ranging from cell therapy to creation of whole new kidneys for organ transplantation [72]. Stem cell sources from the kidney may prove ideal for these applications because of inherent renal differentiation potential and the potential for autologous treatments. With autologous treatments, there is always the concern that diseased organs will have depleted or disfunctional cell sources. While the clinical functionality of these cells will need to be tested for every new cell type, there is at least some evidence showing primary cells from patients with CKD are similar in functionality to normal renal cells [73].

While treatments for CKD are a huge clinical target, most animal models are for acute kidney failure because of the difficulty creating chronic kidney damage. Cell therapy to for acute kidney failure has been mainly with regards to mesenchymal stem cells [74]. Usually limited engraftment is seen and the functional improvement is thought to come from temporary paracrine effects. Renal stem cells may stand a better chance of engraftment in the kidney which may improve the long term healing effects. Toxicity-induced models of acute renal failure are common and well characterized in animals. Adriamycin primarily damages podocytes while glycerol-induced rhabdomyolysis mostly damages tubules and both have been used in animal models for studying renal stem cell treatment [75, 76]. Multiple studies have shown that human CD133 + CD24+ parietal epithelial cells will engraft in toxicity models for acute kidney failure [11, 13, 14]. Results consistently showed morphological recovery from renal damage and engraftment into glomerular and tubular structures, in some cases this engraftment is stable out to 45 days after injury [14]. In addition, functional improvement was seen in all cases reflected in improved BUN and serum creatinine levels or reduced proteinuria. The same cells, but collected from the urine of patients with glomerular disease also showed the ability to engraft and improve function [15]. The number of cells used during treatement and the intravenous delivery method was consistent among the studies, but the number of days after injury varied. Interestingly, the techniques used to isolate a stem cell source originating from parietal epithelium vary slightly, but the ability to improve renal function is consistent. CD133+ cells of tubular origin have also been used to treat toxicity induced acute kidney failure in mice with similar engraftment and functional improvement [11, 53].

Ischemia can also be used to cause acute renal failure. Applying renal stem cells using this model has been more variable, both in the method of model creation, the application of treatement and the results. Kitamura et al injected cells from rat S3 tubule segments into the subcapsular renal space after 40 min ischemia and the removal of one kidney [45]. The cells were found to migrate into the cortex and medulla and integrate into tubules. No functional recovery was noted in either the serum creatinine or BUN. In another case, cells isolated from whole rat kidneys were directly perfused through rat kidneys after 35 min of ischemia and were noted to engraft into tubular structures, but did not show functional improvement [46]. One group isolated what they consider a renal mesenchymal stem cell population from mice. These cells were introduced intravenously to rats after a 25 min ischemic injury and showed renal functional improvement based on serum creatinine and BUN. Interestingly, cell-conditioned media showed the same results [32]. Applications in ischemic injury models have shown less encouraging results than applications in toxicity-induced injury models. This could be because a wider array of cells have been tried to treat ischemia, or it could be from less consistent model creation techniques. The duration of ischemia is tied to the severity of injury and could affect the results [75]. In addition, the severity of injury may be critical to whether certain cells are activated [29]. Ischemic injury in rats may also be more severe and have different underlying mechanisms than ischemic injuries in humans, which may obscure the results further [76].

Tissue engineering offers an additional avenue for utilizing renal stem cells to treat disease. Engineered renal tissue could be used to replace or augment renal function in CKD patients. Renal cells in general have the capacity to form tubule-like structures in 3D culture [77, 78]. The majority of research into 3D tubule formation has been done using cells lines, but tubule formation has also been demonstrated with adult renal cells and non-renal stem cells [77, 78]. Here we will discuss the few examples of 3D tubule formation specifically using possible adult renal stem cells.

Nephrospheres generated from human renal cells were embedded in type 1 collagen and matrigel for 3D culture. In both cases, hollow cysts and tubule-like structures were formed by 10 days [65]. A similar outcome was seen when nephrospheres in collagen were injected subcutaneously in mice. Human CD133+ tubular cells have also been studied in Matrigel to show the formation of polarized tubules [53]. Rat tubule origin cells have been embedded as either 3D clusters in matrigel or as single cells suspended in Type 1 Collagen [26, 69]. In both cases, tubules form, but the outcome was strongly dependent on the growth factors added to the media during culture. Learning how to control the cell culture environment for better tubule formation will be important. One group has employed micropatterned fibronectin strips to show some control over tubule differentation of CD133+ cortical cells [79]. Thinner strips showed effects on cell morphology and improvement in tubule specific marker expression. Research into renal stem cell based 3D culture is still very limited, with no available functional data related to any tubule formation. Additionally, as with all volumetric tissue engineered projects, vascularization will become an issue if renal cell self-assembly is to be applied clinically [80, 81].

## Conclusions

Interest and research into how resident adult stem cell populations in the kidney behave during homeostasis and how they respond to injury have been dramatically increasing recently. Efforts are still scattered among multiple hypothesis regarding the location of any adult stem cells. The most robust cases have been built for populations in the Bowman’s capsule and the base of the papilla, two very different environments. It may be that given the complexity of the kidney that there are multiple cell populations responsible for replacing lost cells. Moving forward, focus will have to be given to consistent methods applied across studies if we are to come to a final understanding of renal adult stem cells. Despite the still early understanding, it seems that most potential sources are highly proliferative in culture, have regenerative capacity, show potential for autologous clinical applications, and remain an enticing option for tissue engineering applications.

## Abbreviations

ALDH: Aldehyde dehydrogenase
APEMP: Adult parietal epithelial multipotent progenitor
BrdU: 5-bromo-2-deoxyuridine
CKD: Chronic kidney disease
EdU: 5-ethynyl-2-deoxyuridine
LRC: Label retaining cell
PEC: Parietal epithelial cell
